# Urodynamic characteristics and clinical significance of detrusor overactivity in early (≤6 months) male urinary incontinence following TURP and RARP: a descriptive analysis of two cohorts

**DOI:** 10.3389/fonc.2026.1902283

**Published:** 2026-08-11

**Authors:** Xiao Zeng, Hong Shen, Deyi Luo, Tao Jin

**Affiliations:** 1 Department of Urology, West China Hospital, Sichuan University, Chengdu, Sichuan, China; 2 Department of Pelvic Floor Diseases Center, West China TianFu Hospital, Sichuan University, Chengdu, Sichuan, China

**Keywords:** International Consultation on Incontinence Questionnaire - Short Form, radical prostatectomy, transurethral resection of the prostate, urinary incontinence, urodynamic study

## Abstract

**Background:**

To describe the urodynamic features of early (≤6 months) male urinary incontinence after transurethral resection of the prostate (TURP) and robot-assisted radical prostatectomy (RARP) as two separate cohorts, and to evaluate the clinical significance of detrusor overactivity (DO) within each group, thereby providing descriptive evidence to support tailored diagnosis and treatment strategies.

**Methods:**

A retrospective analysis was conducted using data from patients diagnosed with early (≤6 months) urinary incontinence (UI) after TURP or RARP. Urodynamic parameters, including maximum cystometric capacity (MCC), first desire to void (FD), strong desire to void (SD), bladder compliance (BC), abdominal leak point pressure (ALPP), and the presence of detrusor overactivity (DO), were analyzed within each cohort.Subgroup analyses based on DO status were performed separately for each group. The severity of UI was assessed using the International Consultation on Incontinence Questionnaire-Short Form (ICI-Q-SF).

**Results:**

A total of 51 patients in the TURP+UI group and 38 patients in the RARP+UI group were included. The TURP+UI group showed significantly higher MCC [260 (207-301) vs 217 (154.50-274.50) mL, P=0.02] and FD (152.78 ± 57.52 vs 123.95 ± 65.65 mL, P=0.03), and lower ICI-Q-SF scores [10 (6-16) vs 19 (17-20), P<0.00] compared to the RARP+UI group. DO was observed in 27.45% of TURP+UI patients and 39.47% of RARP+UI patients. In both groups, patients with DO exhibited significantly lower MCC, FD, SD, and BC values(P<0.00). In the RARP+UI group, patients with DO also had significantly lower ALPP values [0 (0–44) vs 58 (43–91) cmH2O, P<0.001]. Partial correlation analysis revealed that ICI-Q-SF scores were negatively associated with MCC (r=–0.34, p=0.02) and SD (r=[–0.34, p=0.02) in the TURP+UI group, and with ALPP (r=–0.60, p<0.00) in the RARP +UI group.

**Conclusion:**

This study describes the urodynamic characteristics of patients with early (≤6 months) UI following TURP and those with UI after RARP as two separate cohorts. In both groups, the presence of DO was associated with reduced MCC and BC, suggesting that DO is a clinically relevant factor affecting storage-phase function regardless of surgical type. The distinct urodynamic profiles observed between the two cohorts likely reflect differences in underlying pathology and surgical mechanisms. Due to the absence of preoperative urodynamic data, these findings should be interpreted as descriptive phenotypic characterizations rather than comparative evidence.

## 1 Introduction

Prostate cancer (PC) is the most common malignancy in men, according to the latest global prostate cancer survey data, an estimated 313,780 new cases of prostate cancer are projected to be diagnosed in the United States in 2025 (92.29 cases per 100,000 population) (1). In China, the crude incidence rate of prostate cancer is approximately 10.23 cases per 100,000 population (2). Currently, radical prostatectomy (RP) remains the primary treatment option for patients with localized prostate cancer (3). Surgical approaches include open radical prostatectomy (ORP), laparoscopic-assisted radical prostatectomy (LARP), and robot-assisted radical prostatectomy (RARP). In addition to PC, a common benign prostatic condition that affects a large number of middle-aged and elderly men worldwide is benign prostatic hyperplasia (BPH). BPH is defined as an increased number of epithelial and stromal cells in the prostate that can cause lower urinary tract symptoms (LUTS) (4). The global incidence rate of BPH is approximately 326.12 cases per 100,000 population, studies have shown that about 70% of LUTS in male people over 60 years are caused by BPH, seriously affect the quality of life (Qol) of patients (5, 6). BPH-induced chronic bladder outlet obstruction (BOO) triggers progressive bladder remodeling, characterized by detrusor hypertrophy, extracellular matrix accumulation, wall stiffening, and enhanced sensory afferent excitability. These pathological changes predispose to detrusor overactivity (DO), which affects of men with significant BOO and may persist after surgical de-obstruction, thereby contributing to postoperative urgency and urgency incontinence in patients undergoing TURP. In contrast, patients with localized PC generally lack chronic bladder outlet obstruction, as tumors arise in the peripheral zone and seldom compromise the urethral lumen until advanced stages. Therefore, their preoperative detrusor function, including compliance, capacity, remains largely intact. This implies that post-RARP incontinence primarily results from surgical injury, rather than pre-existing bladder pathology, whereas post-TURP incontinence may reflect persistent pre-existing DO combined with *de novo* sphincteric impairment. Current main treatment options include watchful waiting, behavioral therapy, pharmacological treatment, and surgical intervention (7). The current main surgical indications include refractory urinary retention, recurrent urinary tract infections, renal insufficiency, bladder stones, and refractory LUTS that do not respond to other treatments (8).

According to the definition by the International Continence Society (ICS), urinary incontinence (UI) is a condition characterized by the involuntary leakage of urine. It is generally classified into three types: stress urinary incontinence (SUI), urgency urinary incontinence (UUI), and mixed urinary incontinence (MUI). SUI refers to urine leakage that occurs during physical activities such as coughing, sneezing, or moving. UUI is characterized by urine leakage preceded by a sudden, compelling urge to void. MUI involves features of both UUI and SUI (9). Male UI is a significant health concern that profoundly impacts patients’ Quality of life (Qol) and imposes considerable psychosocial, interpersonal, and occupational challenges (10). Male UI most commonly occurs following treatments for PC such as RP or radiotherapy. It may also develop after surgical procedures for BPH, and in some cases, can also occur in patients with neurogenic bladder (NB) (11).

According to previous research, the reported incidence of UI varies by surgical technique, ranging from 7% to 39.5% after OPR, 5% to 33% after LARP, and 4% to 31% following RARP (12). A randomized controlled trial demonstrated superior urinary continence recovery in patients undergoing RALP compared to those undergoing RRP at postoperative intervals of 3, 6, and 18 months (13). The short-term incidence of UI after transurethral resection of the prostate (TURP) is approximately 8%-40% (14, 15). It should be emphasized that factors such as the participating medical centers, surgical techniques employed, surgeons’ experience levels, and follow-up duration in these studies may all contribute to potential bias (16). UI after prostate surgery has been associated with both intrinsic sphincter deficiency (ISD) and bladder dysfunction, with early studies suggesting that detrusor overactivity (DO) was a primary contributing factor (17, 18). Nevertheless, the presence of preoperative OAB or the development of postoperative DO are significant factors to consider (19). The same applies to TURP, where pre-existing OAB prior to surgery can be a cause of persistent postoperative incontinence. Several studies have indicated that surgical damage to pelvic nerves during the procedure may contribute to postoperative bladder dysfunction (20–22). Risk factors for post-prostatectomy UI include higher body mass index (BMI), advanced age, and surgeon experience (20).

Urodynamic study (UDS) is considered the gold standard for evaluating lower urinary tract function (LUTF) (23). Most current studies have confirmed the important role of urodynamic evaluation in the assessment and management of UI following prostatectomy (24–26). However, studies focusing on the urodynamic characteristics of UI following RARP and TURP remain limited. Importantly, patients undergoing these two procedures differ substantially in baseline disease, surgical mechanisms, and pathophysiology of postoperative incontinence. Without preoperative urodynamic data, it is not possible to definitively distinguish between preexisting bladder dysfunction and surgically induced damage as the cause of postoperative findings. Therefore, this study does not aim to compare outcomes between the two surgical approaches. Instead, we describe the urodynamic phenotypes of UI in each cohort separately and explore the clinical significance of DO within each group, with the goal of providing descriptive evidence to support clinical decision-making in the management of post-prostatectomy incontinence.

## 2 Materials and methods

### 2.1 Research design

This study was designed as two parallel descriptive cohort studies, rather than a comparative effectiveness study, due to fundamental differences in patient populations and surgical pathophysiology between the two groups. Patients undergoing TURP and RARP differ not only in surgical technique but also in baseline lower urinary tract function, disease chronicity, and mechanisms of postoperative incontinence. The primary objective of this study is descriptive: to characterize the urodynamic phenotypes of early (≤6 months) UI in each cohort and to explore the clinical significance of DO within each group, rather than to compare outcomes between the two surgical approaches. All between-group comparisons presented in the results are intended as descriptive observations only and should not be interpreted as evidence of procedural superiority. Within each cohort, we performed subgroup analyses stratified by the presence or absence of DO. This allowed us to examine whether DO is associated with differences in storage-phase parameters and whether the pattern of association differs between the two cohorts. These analyses are exploratory and hypothesis-generating, given the absence of preoperative data.

This single retrospective study included data from patients who underwent urodynamic evaluations at West China Hospital of Sichuan University between January 2021 and January 2025. An initial screening was conducted to identify eligible cases. Subsequently, two independent researchers re-evaluated the records based on predefined inclusion and exclusion criteria. Patients were then categorized into two groups according to the type of prostate surgery they had received: Group A (RARP) and Group B (TURP). Relevant urodynamic parameters were analyzed both between the two groups and within each group for subgroup comparisons.

All RARP and TURP procedures included in this study were performed at West China Hospital, Sichuan University, a high-volume tertiary referral center. RARP procedures were performed by a dedicated robotic urology team consisting of three consultant surgeons, each with extensive experience in robotic-assisted surgery and having surpassed the learning curve phase prior to the study period. TURP procedures were performed by four consultant urologists specializing in benign prostatic surgery, each with over 10 years of experience in transurethral surgery. All procedures were performed using standardized surgical techniques according to institutional protocols. No major changes in surgical technique or team composition occurred during the study interval.

### 2.2 Research inclusion criteria

Group A: 1. Clinically and histopathologically diagnosed with prostate cancer via prostate biopsy. 2. UI occurred immediately after surgery or within the first 6 months after the surgery. However, this restriction inherently excludes patients with persistent UI beyond 6 months, who may represent a distinct subgroup with more severe sphincteric or bladder dysfunction. Therefore, our findings specifically reflect the urodynamic characteristics of early (≤6 months) postoperative UI and may not be generalizable to patients with long-standing or persistent incontinence. 3. Underwent postoperative UDS with complete data available. 4. Completed the International Consultation on Incontinence Questionnaire - Short Form (ICI-Q-SF) after surgery. 5. No use of anticholinergic medications or β3-adrenoceptor agonists. 6. No pelvic floor muscle training (PFMT) after surgery. 7. No history of neurological disorders or diabetes. 8. Did not receive postoperative radiotherapy. 9. All patients underwent RARP for prostate cancer.

Group B: 1. Clinically diagnosed with BPH. 2. Normal prostate-specific antigen (PSA) levels. 3. Normal prostate findings on magnetic resonance imaging (MRI). 4. UI occurred immediately after surgery or within the first 6 months after the surgery.5. Complete postoperative UDS data available. 6. Completed the ICI-Q-SF after surgery. 7. No history of neurological disorders or diabetes. 8. No use of anticholinergic medications or β3-adrenoceptor agonists 9. No PFMT after surgery.

### 2.3 Research exclusion criteria

1. History of other pelvic floor surgeries. 2. Duration of UI exceeding 6 months. 3. History of neurological disorders or diabetes. 4. Incomplete UDS data (Incomplete urodynamic data primarily refer to the failure to record all parameters during the patient’s urodynamic study, such as markers during the storage phase like MCC, FD, and SD. This may also include the omission of necessary diagnostic procedures, such as the absence of an abdominal leak point pressure provocation test.). 5. Receipt of any pharmacological or non-pharmacological treatments for UI. 6. Preoperative history of UI.

### 2.4 Urodynamic assessment and research indicators

Urodynamic studies were performed with patients in the seated position, following the guidelines of the ICS and the Good Urodynamic Practice (GUP) protocol. All patients underwent bladder filling with sterile normal saline at 37 °C. The filling rate (mL/min) was set at approximately 10% of the patient’s maximum voided volume, as determined from their voiding diary (27). Therefore, the selected filling rate generally ranges from 30–50 ml/min, with 30 ml/min being the standard rate employed in our experiments to minimize the potential influence attributable to filling rate. Two independent researchers extracted the following parameters from the urodynamic traces: Maximum cystometric capacity (MCC);Volume at First desire to void (FD); Volume at Strong desire to void (SD); Bladder compliance (BC); Abdominal leak point pressure (ALPP): Measured by inducing urinary leakage using the Valsalva maneuver (We performed three Valsalva maneuvers during the bladder filling phase, and the lowest pressure value that induced urine leakage was recorded as the final ALPP. Additionally, particular attention was paid to distinguishing cases of UUI that may have been misclassified as SUI due to DO triggered by the Valsalva maneuver.); Maximum uroflow rate (Qmax); Detrusor pressure at maximum flow rate (Pdet.Qmax); Post-void residual (PVR) volume; Bladder outlet obstruction index (BOOI): BOOI = Pdet.Qmax − 2 × Qmax; Bladder contractility index (BCI): BCI = Pdet.Qmax + 5 × Qmax; Presence and type of detrusor overactivity (DO): Phasic or terminal DO during the filling phase; International Consultation on Incontinence Questionnaire – Short Form (ICI-Q-SF) (This study employed the Chinese version of the ICI-Q-SF scale, which was officially released by the Chinese Urological Association) (28).

### 2.5 Medical ethics

This study received approval from the Medical Ethics Committee of West China Hospital of Sichuan University (No. 2022-1381) and was conducted in strict adherence to the principles outlined in the Declaration of Helsinki. All participants provided written informed consent prior to enrollment.

### 2.6 Statistical analysis

Statistical analysis in this study was conducted using IBM SPSS Statistics for Windows, version 21.0 (IBM Corp., Armonk, NY, USA), and data visualization was carried out using GraphPad Prism version 9.0 (GraphPad Software, San Diego, CA, USA). The Shapiro-Wilk test was employed for assessing the normal distribution of measurement data. If the distribution of numerical data was normal and homogeneous, the student t test was used for inter group differences test, and mean ± standard deviation (SD) was used for statistical description. Conversely, If the distribution of numerical data was skewed or unequal, the Mann-Whitney U rank sum test was used for inter group differences test, and M (P25-P75) was used for statistical description. Pearson chi square test and Fisher’s exact probability were used to analyze the differences in composition between groups. Partial correlation analysis was utilized to examine the correlation between variables. Statistical significance was defined as p< 0.05.

## 3 Results

### 3.1 Baseline characteristics

**Figure 1:**
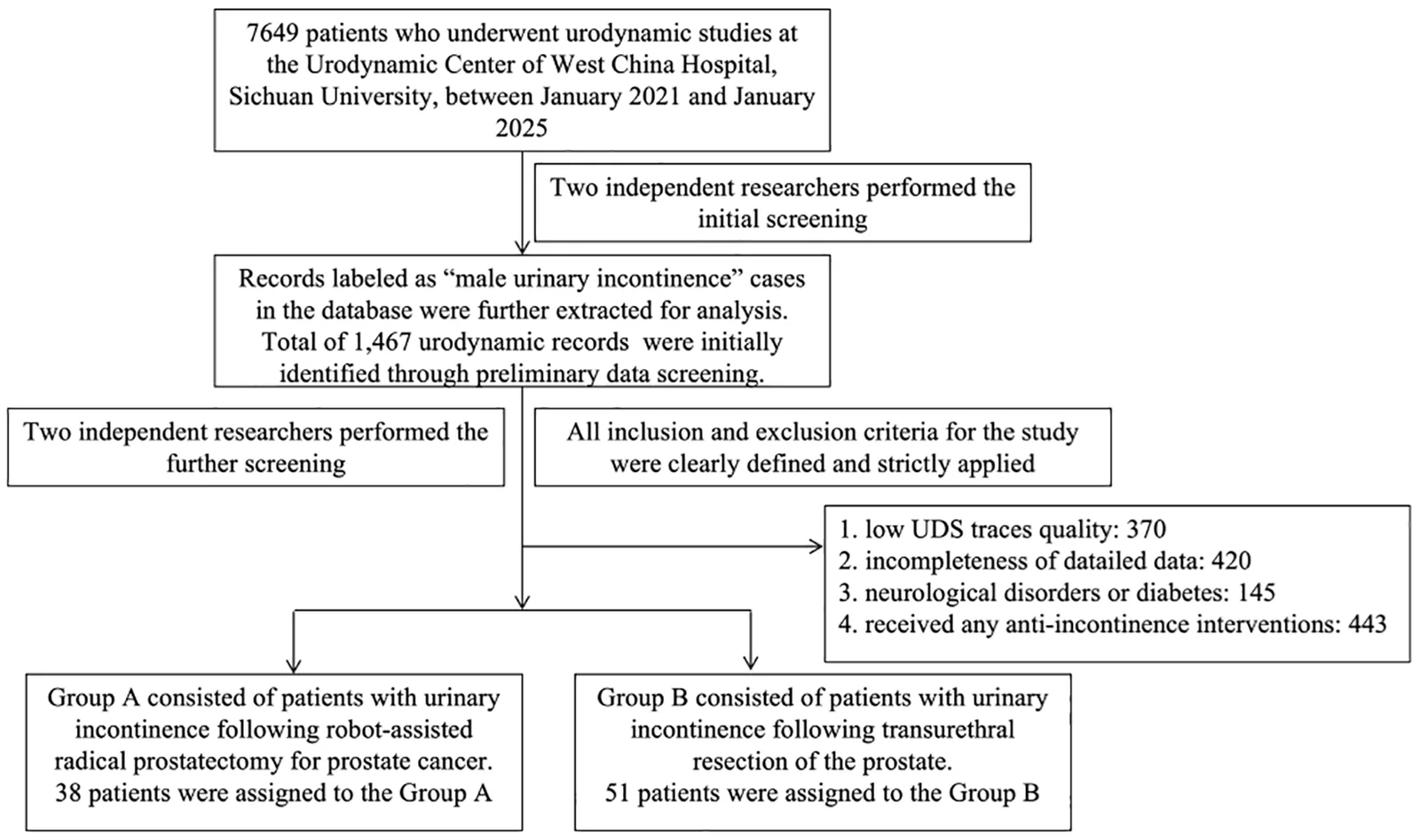
Flowchart of data extraction.

A total of 7,649 urodynamic reports, traces and corresponding patient information were extracted from the database for initial screening within the specified research timeframe. Then, a total of 1,467 urodynamic records labeled as “male urinary incontinence” were initially identified through preliminary data screening. Two independent researchers then re-evaluated the data according to the study’s predefined inclusion and exclusion criteria, as well as the grouping criteria. After rigorous screening, 89 patient records were included in the final analysis. Among them, 38 patients were assigned to Group A, with a median age of 73 (67-75.50) years, and 51 patients to Group B, with a median age of 70 (62-76) years. There was no statistically significant difference in age between the two groups (P=0.19) (**Figure 1**; **Table 1**; **Supplementary Table 1**).

**Table 1:** Comparison of urodynamic measurements and ICI-Q-SF scores between patients with urinary incontinence following RARPand TURP.

Index	RARP+UI	TURP+UI	Z/t	P
Case number	38	51	NA	NA
Age (years)	73 (67-75.50)	70 (62-76)	-1.3	0.19*
MCC (ml)	217 (154.50-274.50)	260 (207-301)	-2.27	0.02*
FD (ml)	123.95 ± 65.65	152.78 ± 57.52	2.2	0.03**
SD (ml)	200 (135-272.50)	240 (200-270)	-1.92	0.06*
BC (ml/cmH2O)	195 (111.25-272.50)	245 (100-300)	-0.58	0.56*
ALPP (cmH2O)	46.5 (0-70.75)	40 (0-76)	-0.23	0.82*
Qmax (ml/s)	4 (1-7.25)	5 (1-10)	-0.78	0.44*
pdet.Qmax (cmH2O)	26.5 (15.75-36)	30 (8-42)	-0.15	0.88*
BCI	49.5 (25.75-71.25)	53 (18-90)	-0.68	0.50*
BOOI	12.5 (5.50-27)	10 (2-27)	-0.45	0.65*
PVR (ml)	65 (3-182.50)	100 (20-200)	-1.44	0.15*
ICI-Q-SF	19 (17-20)	10 (6-16)	-6.06	0.00*

MCC, Maximum bladder cystometry capacity; FD, First desire to void; SD, Strong desire to void; BC, Bladder compliance; ALPP, Abdominal leak point pressure;Qmax, Maximum uroflow rate; pdet.Qmax, Detrusor pressure at maximum flow rate; BCI, Bladder contractility index; BOOI, Bladder outlet obstruction index; PVR, Post residual volume; ICI-Q-SF, International Consultation on Incontinence Questionnaire – Short Form. TURP, Transurethral resection of the prostate; RARP, Robot-assisted radical prostatectomy; *Mann-Whitney U rank sum test; **student t test. NA, Not available.

### 3.2 Results of comparative analysis of parameters between the group A and group B

In Group A, the median MCC was 217 (154.50-274.50) mL, FD was 123.95 ± 65.65 mL, SD was 200 (135-272.5) mL, BC was 195 (111.25-272.5) mL/cmH2O, ALPP was 46.5 (0-70.75) cmH2O, Qmax was 4 (1-7.25) mL/s, Pdet.Qmax was 26.50 (15.75-36) cmH2O, BCI was 49.50 (25.75-71.25), BOOI was 12.50 (5.50-27), PVR was 65 (3-182.50) mL, and the ICI-Q-SF score was 19 (17-20). In Group B, the median MCC was 260 (207-301) mL, FD was 152.78 ± 57.52 mL, SD was 240 (200-270) mL, BC was 245 (100-300) mL/cmH2O, ALPP was 40 (0-76) cmH2O, Qmax was 5 (1-10) mL/s, Pdet.Qmax was 30 (8-42) cmH2O, BCI was 53 (18-90), BOOI was 10 (2-27), PVR was 100 (20-200) mL, and the ICI-Q-SF score was 10 (6,16). There were statistically significant differences between Group A and Group B in terms of MCC [217 (154.50-274.50) vs 260 (207-301) mL, P=0.02], FD (123.95 ± 65.65 vs 152.78 ± 57.52 mL, P=0.03), and ICI-Q-SF score [19 (17-20) vs 10 (6-16), P<0.001] (**Table 1**).

### 3.3 Characteristics of DO and urinary incontinence subtypes of the two groups

In Group A,DO was observed in 15 patients (39.47%), while in Group B, 14 patients (27.45%) exhibited DO. There was no statistically significant difference in the incidence of DO between the two groups (P = 0.23). Regarding DO subtypes, in Group A, there were 11 cases of TDO and 4 cases of PDO; whereas Group B, there were 10 cases of TDO and 4 cases of PDO. The distribution of DO subtypes between the two groups showed no significant difference (P = 1.00). In terms of UI types (urodynamic diagnosis), in Group A, there were 4 cases of MUI (10.53%), 23 cases of SUI (60.53%), and 11 cases of UUI (28.94%). Group B included 8 cases of MUI (5.69%), 37 cases of SUI (72.55%), and 6 cases of UUI (11.76%). No statistically significant differences were found between the two groups in the distribution of UI types (P = 0.12) (**Table 2**).

**Table 2:** Analysis of detrusor overactivity related urodynamic features and their differences in patients with urinary incontinence following RARP and TURP.

Index		RARP+UI	TURP+UI	chisq	P*
Whether has DO	No	23(60.53)	37(72.55)	1.43	0.23
	Yes	15(39.47)	14(27.45)		
DO type	TDO	11(28.95)	10(19.61)		1.00
	PDO	4(10.53)	4(7.84)		
UI type**	MUI	4(10.53)	8(15.69)	4.26	0.12
	SUI	23(60.53)	37(72.55)		
	UUI	11(28.95)	6(11.76)		

TURP, Transurethral resection of the prostate; RARP, Robot-assisted radical prostatectomy; DO, Detrusor overactivity; UI, Urinary incontinence; MUI, Mixed urinary incontinence; SUI, Stress urinary incontinence; UUI, Urgency urinary incontinence; TDO, Terminal detrusor overactivity; PDO, phasic detrusor overactivity; *Fisher's exact probability; **Urodynamic diagnosis.

### 3.4 A subgroup analysis was conducted in patients with RARP urinary incontinence based on the presence or absence of DO

**Figure 2:**
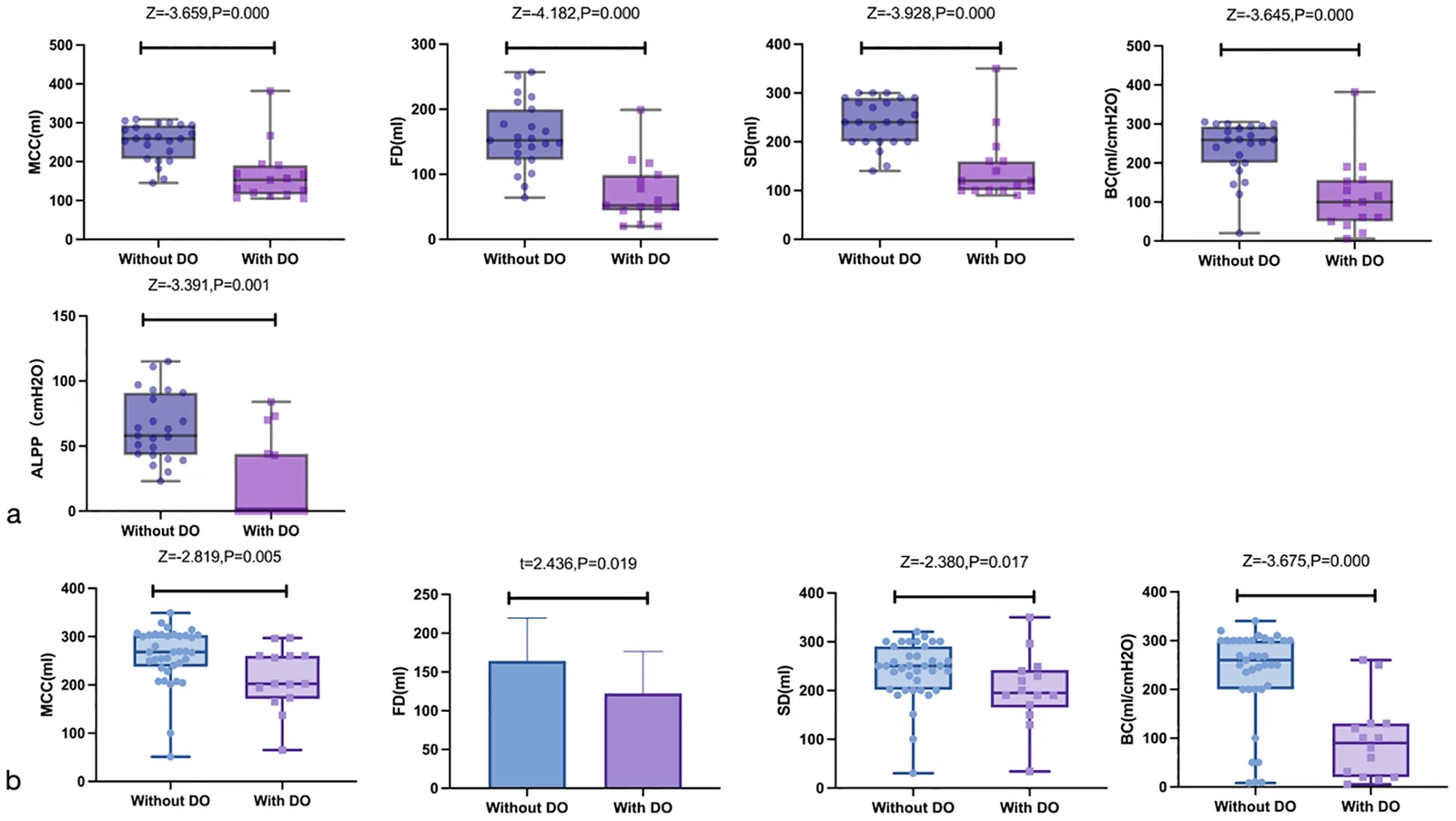
Urodynamic characteristics of patients with urinary incontinence after TURP and RARP, stratified by the presence or absence of detrusor overactivity (DO). **(a)** Comparison of urodynamic parameters between urinary incontinence patients with and without detrusor overactivity during urodynamic study after RARP **(b)** Comparison of urodynamic parameters between urinary incontinence patients with and without detrusor overactivity during urodynamic study after TURP.

**Figure 3:**
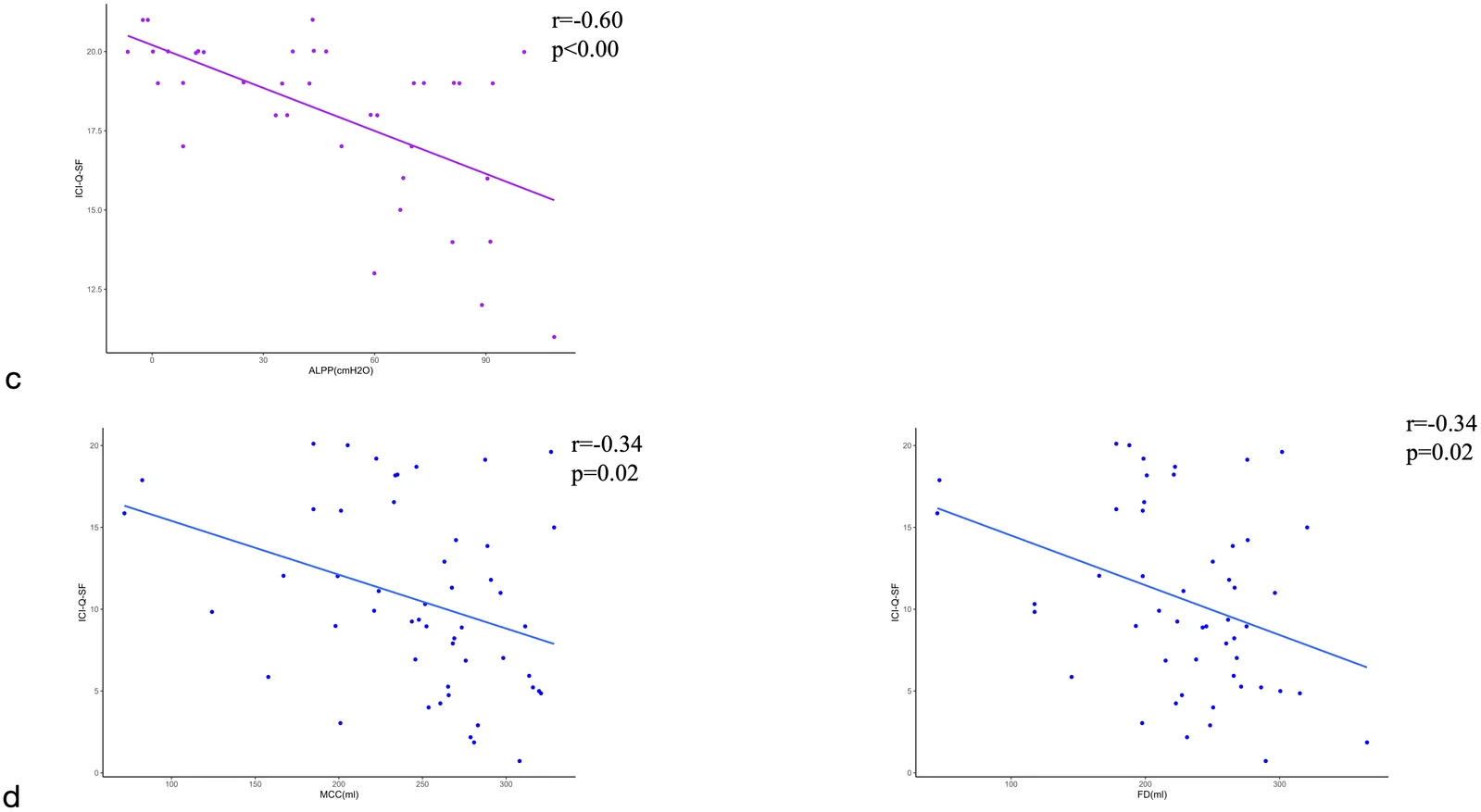
Partial correlation analysis between ICI-Q-SF scores and urodynamic parameters. **(c)** Partial correlation analysis between ICI-Q-SF scores and urodynamic parameters in patients with urinary incontinence after RARP. **(d)** Partial correlation analysis between ICI-Q-SF scores and urodynamic parameters in patients with urinary incontinence after TURP.

In Group A, DO was observed in 23 patients, while 15 patients did not exhibit DO. There was no statistically significant difference in age between the two subgroups (P = 0.63). However, significant differences were found in several urodynamic parameters. The median MCC was significantly higher in the non-DO group compared to the DO group [259 (207–294) vs 153 (115–191) mL, p<0.001]. Similarly, FD [152 (122–200) vs 52 (44–99) mL, p<0.001], SD [240 (200–290) vs 120 (100–160) mL, P<0.001], BC [259 (200–294) vs 100 (50–157) mL/cmH2O, P<0.001], and ALPP [58 (43–91) vs 0 (0–44) cmH2O, P = 0.00] were all significantly higher in the non-DO group. No significant differences were observed in the remaining parameters between the two subgroups. In the partial correlation analysis between ICI-Q-SF scores and urodynamic parameters (after controlling for age), a significant negative correlation was observed between ICI-Q-SF and ALPP (r= –0.60, p<0.00), while no significant correlations were found with the other parameters (**Figures 2a**, **3c**; **Tables 3**, **4**).

**Table 3:** Subgroup analysis of urodynamic and ICI-Q-SF characteristics in Post-RARP urinary incontinence patients with and without detrusor overactivity.

Index	DO presence Yes or DO presence No		Z/t	P
	No	Yes		
Case number	23	15	NA	NA
Age (years)	74 (67-75)	72 (67-78)	-0.48	0.63*
MCC (ml)	259 (207-294)	153 (115-191)	-3.66	0.00*
FD (ml)	152 (122-200)	52 (44-99)	-4.18	0.00*
SD (ml)	240 (200-290)	120 (100-160)	-3.93	0.00*
BC (ml/cmH2O)	259 (200-294)	100 (50-157)	-3.65	0.00*
ALPP (cmH2O)	58 (43-91)	0 (0-44)	-3.39	0.00*
Qmax (ml/s)	4 (1-8)	4 (1-6)	-0.78	0.44*
pdet.Qmax (cmH2O)	25.04 ± 15.24	27.47 ± 20.12	-0.42	0.68**
BCI	52.22 ± 37.3	46.80 ± 29.43	0.47	0.64**
BOOI	12 (6-22)	21 (2-27)	-0.57	0.57*
PVR (ml)	120 (12-200)	46 (0-120)	-1.31	0.19*
ICI-Q-SF	19 (15-20)	19 (18-20)	-1.72	0.09*

MCC, Maximum bladder cystometry capacity; FD, First desire to void; SD, Strong desire to void; BC, Bladder compliance; ALPP, Abdominal leak point pressure;Qmax, Maximum uroflow rate; pdet.Qmax, Detrusor pressure at maximum flow rate; BCI, Bladder contractility index; BOOI, Bladder outlet obstruction index; PVR, Post residual volume; ICI-Q-SF, International Consultation on Incontinence Questionnaire – Short Form. TURP, Transurethral resection of the prostate; RARP, Robot-assisted radical prostatectomy; DO, Detrusor overactivity; *Mann-Whitney U rank sum test; **student t test. NA, Not available.

**Table 4:** Partial correlation analysis between ICI-Q-SF scores and urodynamic parameters in patients with urinary incontinence after RARP and TURP.

Index	ICI-Q-SF (RARP group)		ICI-Q-SF (TURP group)	
	r	P*	r	P*
MCC(ml)	-0.23	0.17	-0.34	0.02
FD(ml)	-0.22	0.19	-0.25	0.08
SD(ml)	-0.27	0.11	-0.34	0.02
BC(ml/cmH2O)	-0.20	0.23	-0.17	0.25
ALPP(cmH2O)	-0.60	0.00	0.23	0.12
Qmax(ml/s)	-0.15	0.38	-0.18	0.22
pdet.Qmax(cmH2O)	-0.12	0.47	-0.23	0.12
BCI	-0.16	0.34	-0.22	0.12
BOOI	-0.05	0.76	-0.2	0.17
PVR(ml)	-0.13	0.46	0.07	0.65

MCC, Maximum bladder cystometry capacity; FD, First desire to void; SD, Strong desire to void; BC, Bladder compliance; ALPP, Abdominal leak point pressure;Qmax, Maximum uroflow rate; pdet.Qmax, Detrusor pressure at maximum flow rate; BCI, Bladder contractility index; BOOI, Bladder outlet obstruction index; PVR, Post residual volume; ICI-Q-SF, International Consultation on Incontinence Questionnaire – Short Form. RARP, Robot-assisted radical prostatectomy; *Partial correlation analysis.

### 3.5 A subgroup analysis was conducted in patients with TURP urinary incontinence based on the presence or absence of DO

In Group B, DO was observed in 37 patients, while 14 patients did not exhibit DO. There was no statistically significant difference in age between the two subgroups (P = 0.12). However, significant differences were found in several urodynamic parameters. The median MCC was significantly higher in the DO group compared to the non-DO group [268 (237.50–303) vs 202 (171–260) mL, p=0.00]. Similarly, FD [164.30 ± 55.09 vs 122.36 ± 54.29 mL, p=0.02], SD [250 (201–290) vs 195 (165–254.25) mL, P = 0.02], BC [260 (200–300) vs 90 (20–130) mL/cmH2O, P<0.001] were all significantly higher in the DO group. No significant differences were observed in the remaining parameters between the two subgroups. Further partial correlation analysis (after controlling for age) revealed that ICI-Q-SF scores were negatively correlated with MCC (r=–0.34, p=0.02) and SD (r=–0.34, p=0.02). No significant correlations were found between ICI-Q-SF and the remaining urodynamic parameters (**Figures 2b**, **3d**; **Tables 5**, **4**).

**Table 5:** Subgroup analysis of urodynamic and ICI-Q-SF characteristics in Post-TURP urinary incontinence patients with and without detrusor overactivity.

Index	DO presence Yes or DO presence No		Z/t	P
	N	Y		
Case number	37	14	NA	NA
Age (years)	68.70 ± 8.04	72.71 ± 8.25	-1.580	0.12**
MCC (ml)	268 (237.50-303)	202 (171-260)	-2.820	0.01*
FD (ml)	164.30 ± 55.09	122.36 ± 54.29	2.440	0.02**
SD (ml)	250 (201-290)	195 (165-242.25)	-2.38	0.02*
BC (ml/cmH20)	260 (200-300)	90 (20-130)	-3.680	0.00*
ALPP (cmH2O)	57 (0-84.50)	15 (0-58.75)	-1.90	0.06*
Qmax (ml/s)	6 (0.50-11)	3 (0.75-6.25)	-1.13	0.26*
pdet.Qmax (cmH2O)	30 (4-41)	26.5 (7.50-48)	-0.23	0.82*
BCI	70 (9-91.50)	41 (15-79)	-0.70	0.48*
BOOI	10 (1-22.50)	19.5 (4.50-35)	-1.16	0.25*
PVR (ml)	80 (20-220)	165 (55-200)	-1.10	0.27*
ICI-Q-SF	9 (5-14.50)	12 (8.25-17.25)	-1.05	0.30*

MCC, Maximum bladder cystometry capacity; FD, First desire to void; SD, Strong desire to void; BC, Bladder compliance; ALPP, Abdominal leak point pressure;Qmax, Maximum uroflow rate; pdet.Qmax, Detrusor pressure at maximum flow rate; BCI, Bladder contractility index; BOOI, Bladder outlet obstruction index; PVR, Post residual volume; ICI-Q-SF, International Consultation on Incontinence Questionnaire – Short Form. TURP, Transurethral resection of the prostate; RARP, Robot-assisted radical prostatectomy; DO, Detrusor overactivity; *Mann-Whitney U rank sum test; **student t test. NA, Not available.

## 4 Discussions

Male UI significantly impairs patients’ Qol and imposes a substantial economic burden on both individuals and the healthcare system (11). Previous studies have clearly identified prostate surgery as a major cause of male UI. For instance, it has been reported that the incidence of SUI at one year following RARP for PC is approximately 20%, whereas the incidence drops to 1.4% at ten years post-surgery (13). Other studies have also confirmed that the majority of incontinence cases emerge within the first three months after surgery, and approximately one-third of patients continue to exhibit symptoms of UI at one year postoperatively (21, 29). Male urinary continence is maintained by two anatomically distinct components: the internal and external urethral sphincters. The overall continence mechanism also relies on intact neural pathways and supportive pelvic structures. Disruption of these components—such as pelvic nerve injury, impairment of the sphincteric apparatus, or the presence of DO, can all lead to the development of UI in men (16, 30).

As age is a well-established risk factor for post-prostatectomy UI, we first analyzed the age distribution of patients with incontinence following TURP and RARP included in this study (31). The results showed no statistically significant difference in age between the two groups. In further analyses, we found that the TURP+UI group exhibited significantly higher MCC and FD compared to the RARP+UI group [MCC: 260 (207-301) mL vs 217 (154.50-274.50) mL, p=0.02]; FD: [152.78 ± 57.52 mL vs 123.95 ± 65.65 mL, p=0.03]. Additionally, the TURP+UI group had significantly lower ICI-Q-SF scores than the RARP+UI group [10 (6-16) vs 19 (17-20), p<0.001], indicating that the severity of UI was lower in the TURP group compared to the RARP group. The higher MCC observed in the TURP+UI group suggests relatively good bladder storage function. This finding implies that the actual volume of urine leakage due to incontinence may be smaller in this group, which is consistent with the significantly better ICI-Q-SF scores observed in the TURP+UI group. However, no significant difference was observed between the two groups in terms of ALPP [40 (0-76) cmH2O vs 46.5 (0-70.75) cmH2O, p=0.82], which reflects the function of the intrinsic sphincter function. It is generally recognized that an ALPP value below 20 cmH2O indicates the presence of intrinsic sphincter deficiency (ISD). Current studies have confirmed that damage to the IS is the primary cause of post-prostatectomy UI, with a reported incidence ranging from approximately 67% to 92.4% (32, 33). The findings of this study suggest that there may be no significant difference in the extent of IS damage between the two surgical approaches. However, further studies with larger sample sizes are needed to validate this observation.

Bladder dysfunction after RP may result from a variety of mechanisms, including: (a) nerve pathway disruption due to displacement affecting both autonomic and somatic innervation; (b) inflammation of the bladder; (c) structural alterations of the bladder wall potentially caused by chronic preoperative hypoxia (34). However, it should be noted that these mechanisms remain largely speculative, as most evidence is derived from animal models or indirect clinical observations. Moreover, the development of anastomotic strictures and subsequent urinary obstruction following RARP can be contributing factors in the onset of overactive bladder (OAB) symptoms with DO (22, 34). Research indicates that the prevalence of DO is approximately 10% in the general population, while in elderly populations, its incidence can reach as high as 80%. DO primarily manifests as symptoms such as urinary frequency, urgency, and incontinence, which can severely impair patients’ quality of life (35). DO is a key pathophysiological mechanism underlying UUI. In this study, DO was observed in 14 patients (27.45%) in the TURP+UI group and 15 patients (39.47%) in the RARP+UI group, with no statistically significant difference between the groups (p=0.23). Furthermore, based on urodynamic assessments combined with clinical symptoms, the incidence of SUI, UUI, and MUI in the TURP+UI group were 72.5%, 11.76%, and 15.69%, respectively. In the RARP+UI group, the corresponding incidences were 60.53%, 28.94%, and 10.53%, respectively. There was no statistically significant difference in the distribution of these incontinence subtypes between the two groups (p=0.12). In both groups, SUI demonstrated the highest incidence among all UI subtypes. This finding suggests that clinicians should pay particular attention to the prevention and treatment of SUI following both types of prostate surgeries, such as initiating early pelvic floor rehabilitation training. HoLEP, a laser-based enucleation technique for BPH, differs from TURP in its energy source, tissue removal mechanism, and hemostatic properties. While both procedures are used for surgical treatment of BPH, their different tissue effects—including the extent of thermal injury and depth of tissue penetration—may influence postoperative UI mechanisms and recovery patterns. Therefore, the UI rates reported after HoLEP should be extrapolated to TURP with caution. A study on HoLEP reported that within one month postoperatively, 20.8% of patients presented with pure UUI, while 12.7% exhibited SUI; at six months, these figures declined to 4% and 4%, respectively. Whether these rates are directly applicable to TURP patients remains unclear and warrants further comparative investigation (31).

Following RP for PC, reported rates of DO vary significantly among studies. A Japanese study found that approximately 37.8% of patients exhibited DO at three months postoperatively. In contrast, a Spanish study reported a much lower incidence of 9.3%, while a study conducted by Chinese researchers found a DO incidence of 11.8% (19, 36, 37). These findings suggest that the occurrence of UUI is evident following both TURP and RARP. However, the current researches cannot confirm whether DO is caused by the surgery or whether the patient already had DO before the surgery, this still requires further experiments to verify. Therefore, this clinical manifestation should be given due attention during evaluation and management. Taken together, these observations indicate that postoperative patients with UI demonstrated differing urodynamic profiles; however, whether these findings are attributable to surgery itself cannot be determined from the present data. The absence of preoperative urodynamic assessment precludes any causal attribution of postoperative DO to surgical damage versus preexisting bladder pathology. Future prospective studies with preoperative UDS and longitudinal follow-up are needed to clarify the natural history of bladder dysfunction in patients undergoing TURP and RARP, and to distinguish between preexisting conditions and surgically induced changes. Antimuscarinic agents or β3-adrenoceptor agonists may be considered to suppress DO, thereby helping to control symptoms of UUI or MUI.

Subgroup analyses were subsequently conducted within each surgical group based on the presence or absence of DO observed UDS. In the TURP+UI group, patients with DO (14/51) demonstrated significantly lower values of MCC, FD, SD, and BC compared to those without DO. However, no statistically significant difference was observed between the two subgroups in terms of ALPP or ICI-Q-SF scores. with higher scores indicating greater severity of urinary incontinence. The difference in MCC may be attributed to UUI caused by DO, which in turn leads to reduced bladder storage capacity. Additionally, DO increases bladder sensory sensitivity, which may explain the observed differences in both FD and SD values (38). Further correlation analysis within this subgroup revealed a negative relationship between ICI-Q-SF scores and both MCC and SD values. This indicates that lower MCC and SD are associated with worse incontinence scores, reflecting increased severity and greater patient distress related to urinary incontinence symptoms. This is primarily because DO, which leads to reductions in MCC and FD, exacerbates UI symptoms as well as frequency and urgency. Consequently, DO further diminishes patients’ Qol by intensifying the burden of urinary symptoms. Specifically, the ALPP values were significantly lower in the DO subgroup compared to the non-DO subgroup. This may suggest that patients without DO in the RARP+UI group have less impairment of intrinsic urethral sphincter function. Further partial correlation analysis (after controlling for age) within this group revealed a significant negative correlation between ICI-Q-SF scores and ALPP. Since lower ALPP values indicate a higher likelihood of ISD, this is associated with more severe UI, which in turn results in worse ICI-Q-SF scores. Further analysis of DO subtypes within the two cohorts reveals that in the RARP group, the incidence rate of terminal detrusor overactivity (TDO) was 28.95%, while the incidence rate of phasic detrusor overactivity (PDO) was 10.53%. In the TURP group, the incidence rate of TDO was 19.61%, and that of PDO was 7.84%. No statistically significant differences in incidence rates were observed between the two groups. Current research indicates that patients developing TDO manifest more severe symptoms compared to those with PDO, along with inferior responses to both pharmacologic and surgical treatments. Analysis of patient DO subtypes can provide further guidance for physicians in selecting treatment regimens and predicting patient prognosis (39, 40). Two important confounders should be considered. First, the relative contributions of ISD and DO differ between groups: RARP-related UI is predominantly sphincteric, whereas TURP-related UI more often involves preexisting DO. This mechanistic divergence complicates direct comparisons of urodynamic parameters. Second, DO may be *de novo* (surgery-induced in RARP) or persistent (preexisting from BOO in TURP), which has distinct therapeutic implications. Without preoperative UDS, our study cannot fully differentiate these, highlighting a key limitation of retrospective analysis.

One of the most noteworthy findings of this study is the observation that, in the RARP+UI cohort, patients with DO exhibited significantly lower ALPP values compared to those without DO. By contrast, this association was not observed in the TURP+UI cohort. This differential pattern suggests a synergistic interaction between DO and sphincteric dysfunction that appears to be specific to the RARP setting, and raises important questions about the underlying pathophysiological mechanisms. Anatomically, RARP involves dissection of the neurovascular bundles and division of the urethra, potentially affecting both the autonomic and somatic pelvic innervation. TURP, in contrast, is confined to the prostatic urethra and transitional zone, with minimal disruption of somatic sphincteric innervation. We hypothesize that the coexistence of DO and low ALPP in the RARP cohort reflects a more extensive pattern of pelvic nerve injury involving both autonomic and somatic pathways—a “combined neurogenic bladder” phenotype that represents a more severe form of neurogenic dysfunction than either condition alone (14, 17, 22).

Several limitations should be acknowledged when interpreting the findings of this study. First, and most importantly, the absence of preoperative urodynamic data prevents us from definitively distinguishing between: (a) preexisting bladder dysfunction that was present before surgery and either persisted or was unmasked postoperatively; and (b) surgically induced dysfunction that developed as a direct consequence of the procedure. This limitation directly constrains our ability to answer the fundamental question of whether postoperative UI primarily reflects preexisting pathology or surgical damage. Second, because patients in the TURP and RARP cohorts differ in underlying disease, surgical mechanisms, and baseline lower urinary tract function, any between-group comparisons are subject to confounding and should not be interpreted as evidence of procedural superiority. This study was not designed for such comparative purposes. Third, the sample size is relatively small, and no formal sample size calculation was performed prior to the study due to its retrospective nature and the strict inclusion and exclusion criteria, which may limit statistical power and generalizability. Our findings are specific to patients with early postoperative UI who underwent UDS for clinical indications and may not be generalizable to all patients undergoing TURP or RARP, nor to those with chronic, persistent UI. Fourth, the retrospective design may introduce selection bias and limit the ability to establish causal relationships. Some potentially relevant confounding factors, such as variations in surgical techniques, postoperative rehabilitation protocols, and patient comorbidities, were not fully controlled or analyzed. Fifth, the classification of UI was based on urodynamic findings rather than patient-reported symptoms, and reliance on retrospective medical records may result in underestimation or misclassification of clinical diagnoses. Finally, the assessment of urinary symptoms relied partly on patient-reported questionnaires, which may be subject to recall bias and subjective interpretation.

## 5 Conclusions

This study describes the urodynamic characteristics of patients with early (≤6 months) UI following TURP and those with UI after RARP as two separate cohorts. In both groups, the presence of DO was associated with reduced MCC and BC, suggesting that DO is a clinically relevant factor affecting storage-phase function regardless of surgical type. The distinct urodynamic profiles observed between the two cohorts, particularly the association of DO with lower ALPP in the RARP group, may reflect differences in underlying pathophysiology, including the relative contributions of preexisting bladder dysfunction versus surgically induced damage. However, these interpretations remain hypothesis-generating. Prospective studies with preoperative urodynamic assessment are needed to further elucidate the causal pathways of post-prostatectomy incontinence and to guide individualized management strategies. However, the most important caveat of this study is that patients in the TURP and RARP cohorts differ fundamentally in underlying disease, surgical extent, and baseline lower urinary tract function. Consequently, any observed differences in urodynamic parameters between the two groups are highly likely to be attributable to these inherent disparities rather than to differential effects on the continence mechanism per se. Whether these differences represent preexisting bladder dysfunction, surgically induced changes, or a combination of both cannot be determined from the present data.

## Data Availability

The raw data supporting the conclusions of this article will be made available by the authors, without undue reservation.
